# Supplementary figures and images for: Plasma Trimethylamine-N-oxide and impaired glucose regulation: Results from The Oral Infections, Glucose Intolerance and Insulin Resistance Study (ORIGINS)
Source: PLoS One. 2020 Jan 15;15(1):e0227482. doi: 10.1371/journal.pone.0227482 (PMC6961885; doi:10.1371/journal.pone.0227482)

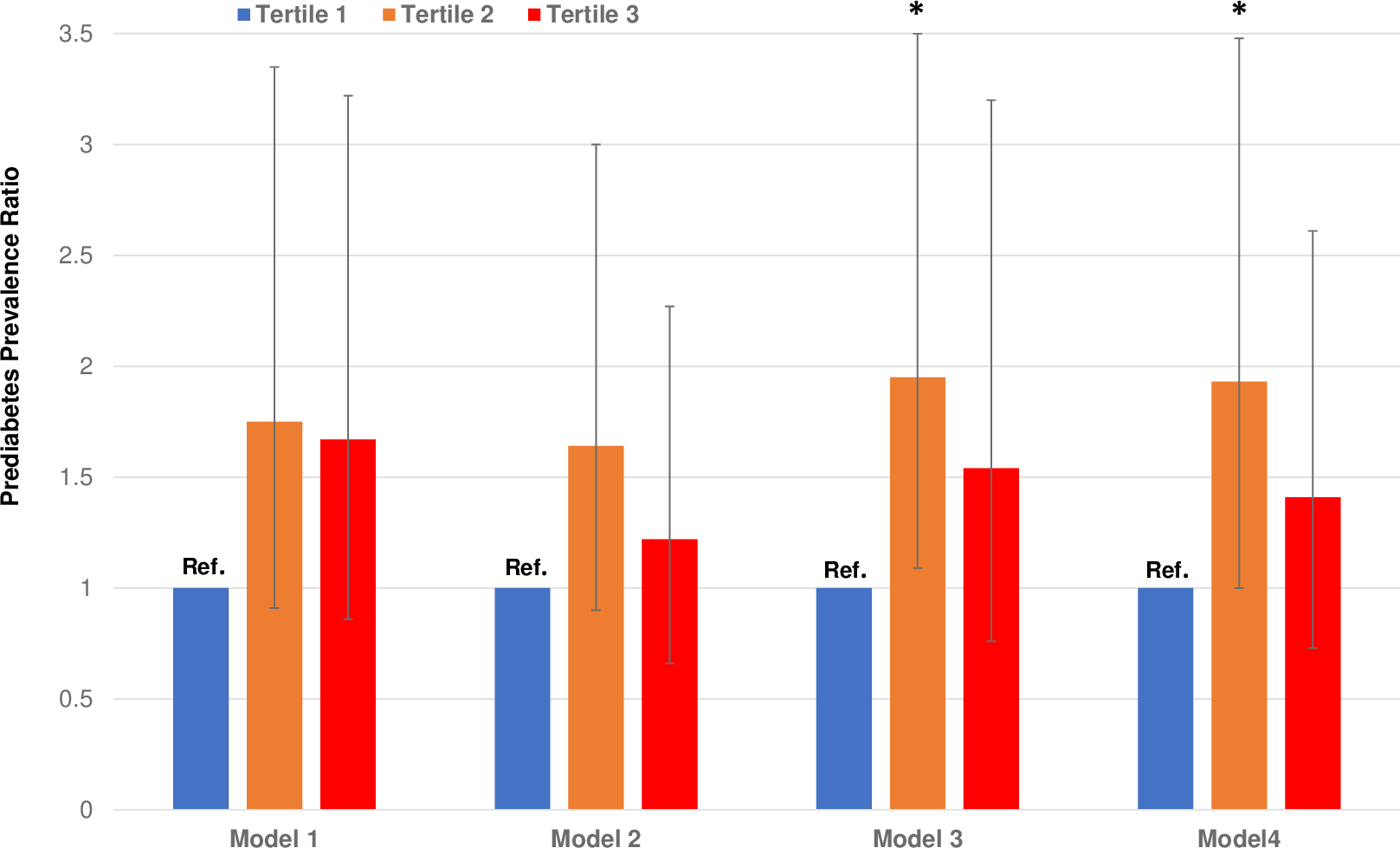

Supplement: S1 Fig — Model 1 = unadjusted; Model 2 = age, gender, race/ethnicity, education; Model 3 = M2+ BMI, systolic blood pressure, HDL; Model 4 = M3+alternative healthy eating index. *p<0.05. (TIF) [file pone.0227482.s001.tif]

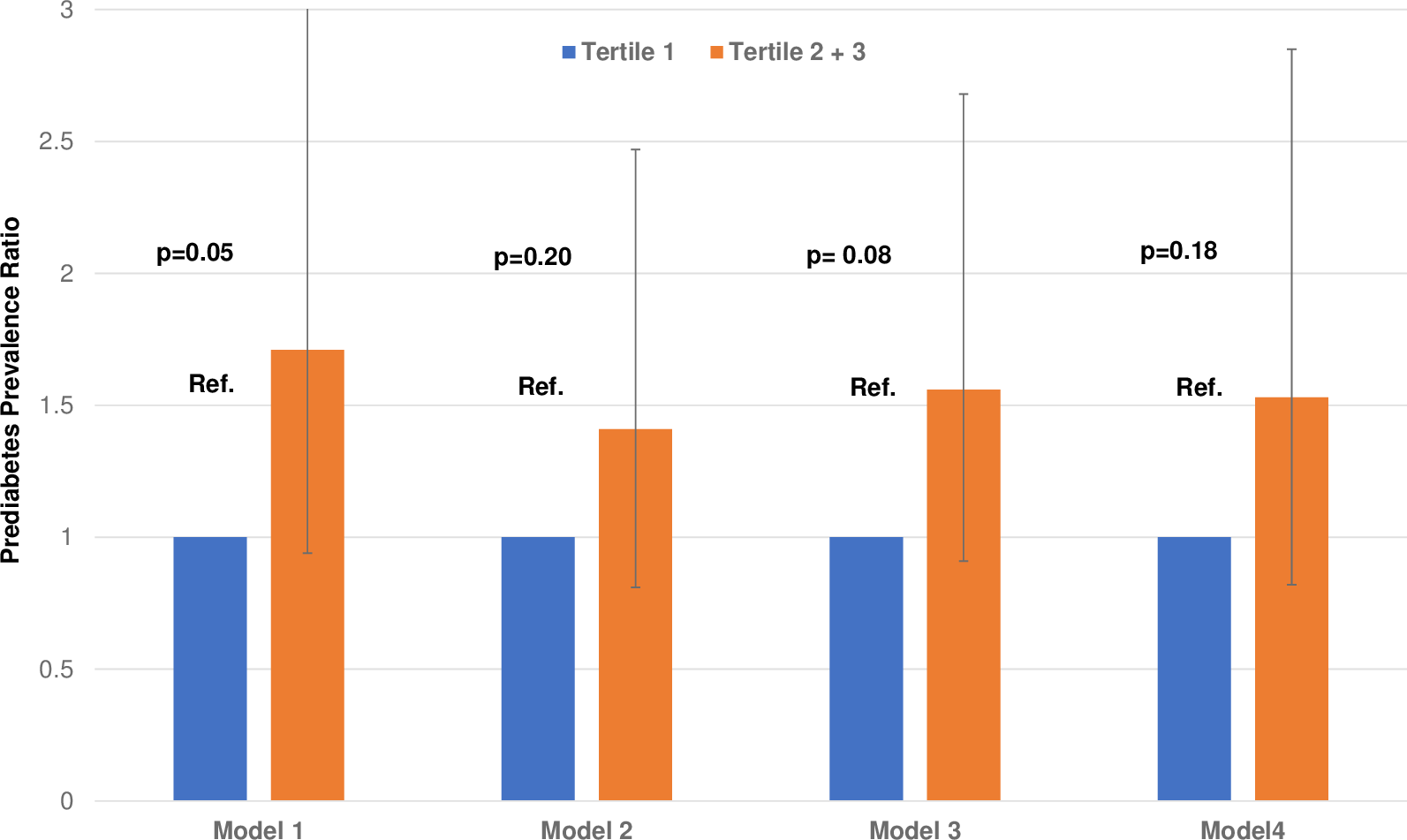

Supplement: S2 Fig — Model 1 = unadjusted; Model 2 = age, gender, race/ethnicity, education; Model 3 = M2+ BMI, systolic blood pressure, HDL; Model 4 = M3+ alternative healthy eating index. (TIF) [file pone.0227482.s002.tif]

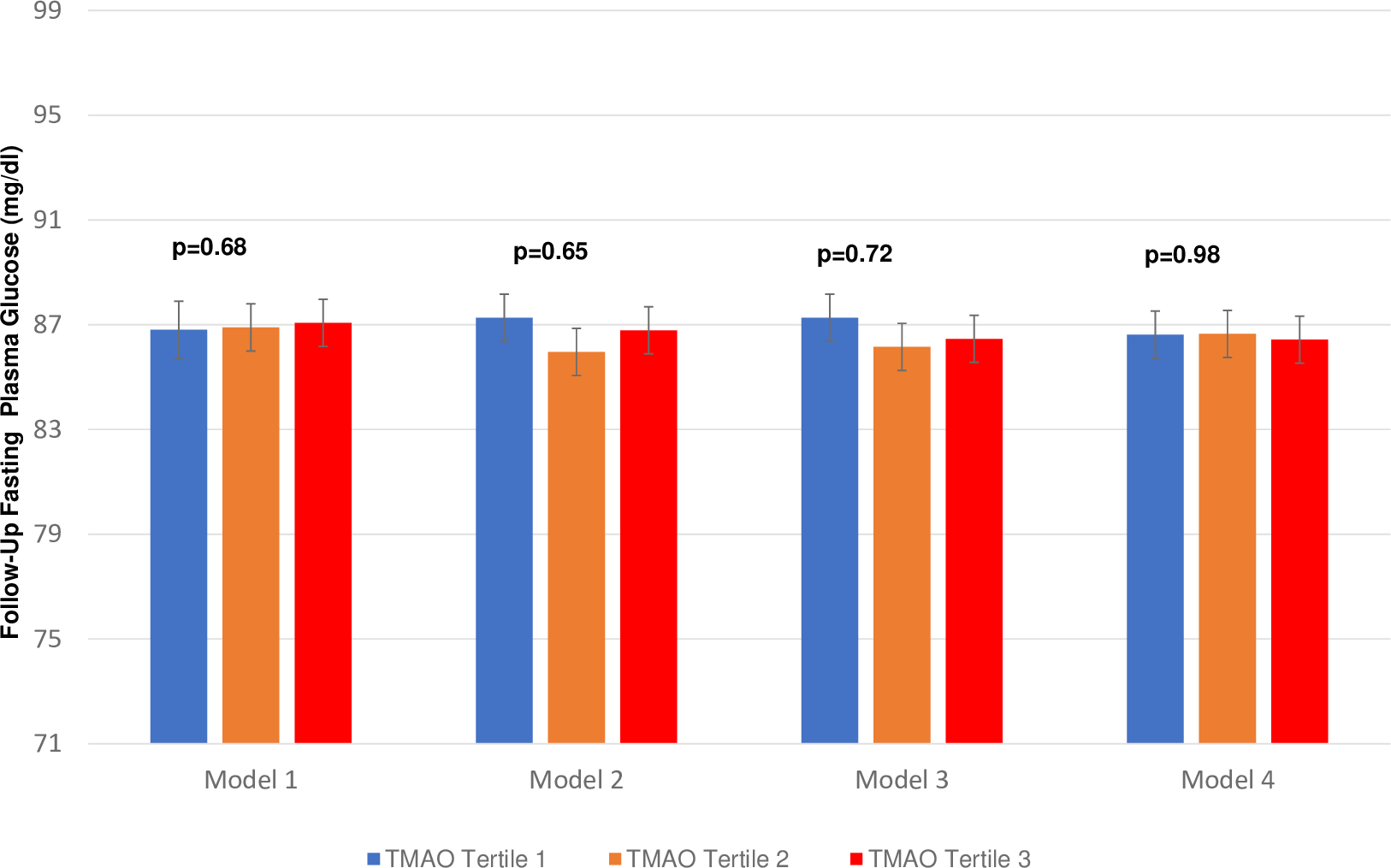

Supplement: S3 Fig — Model 1 = unadjusted; Model 2 = age, gender, race/ethnicity, education; Model 3 = M2+BMI, systolic blood pressure, HDL+ alternative healthy eating index; Model 4 = M3+baseline glucose. Y axis is centered on the mean value observed in the total population (mean = 85 mg/dL) and the range is set to twice the standard deviation of fasting glucose. (TIF) [file pone.0227482.s003.tif]
