# Supplementary material for: Plasma Trimethylamine-N-oxide and impaired glucose regulation: Results from The Oral Infections, Glucose Intolerance and Insulin Resistance Study (ORIGINS)
Source: PLoS One. 2020 Jan 15;15(1):e0227482. doi: 10.1371/journal.pone.0227482 (PMC6961885; doi:10.1371/journal.pone.0227482)
Supplement: Attachment — Submitted filename: Response to Reviewers.docx [file pone.0227482.s004.docx]

We wish to thank the reviewers for their helpful comments, which have led to further strengthening of the manuscript. We provide a point-by-point response to reviewer comments below.

**Reviewer #1 Comments**

**General comments**: Roy and colleagues present the relationship between plasma TMAO and early biomarkers of type 2 diabetes risk from ORIGINS based on the participants who are men and women aged 20-55 years, diabetes-free populations without a history of cardiovascular and/or kidney disease. The results are not hugely ground-breaking. However, overall, the manuscript is technically sound and the results are well interpreted.

**Response**: We appreciate the reviewer’s comments and have responded to each specific comment below in a point-by-point fashion.

**Comment #1**: The authors need to provide the rationale to use the methods and statistical analysis in the body to appreciate why they were done (eg. why do the authors do a two-year follow-up visit to measure TMAO? Why do they use UPLC-MS/MS to measure TMAO?)

**Response**: We wish to clarify that TMAO was only measured once at baseline in this study. This is stated on page 6 (line 117) of our revised submission.

In this analysis, we were interested in investigating the following questions:

a. Is baseline plasma TMAO levels associated with insulin resistance, fasting glucose levels and HbA1c cross-sectionally
b. Is baseline plasma TMAO levels associated with longitudinal increase in fasting plasma glucose
c. Is baseline plasma TMAO levels are associated with increased prediabetes prevalence cross-sectionally

To our knowledge there are no data addressing the association between baseline TMAO and longitudinal fasting plasma glucose among generally healthy, diabetes-free population.

Traditional assays for choline metabolites such as TMAO, including radioenzymatic assays, liquid chromatography with electrochemical detection and GCMS are characterized by low specificity and tedious sample preparation with multi-step derivatization protocols.  Targeted metabolomic approaches employing Liquid Chromatography-tandem mass spectrometry (UPLC-MS/MS) platforms under multiple reaction mode offer high sensitivity, specificity, accuracy, and precision for the quantitation of small molecules. The UPLC-MS/MS based assay used in the present study enabled the sensitive and accurate measurement of TMAO in the human samples. The assay has a lower limit of quantitation (LLOQ) of 0.05uM. The intra-assay accuracy and precision 98% and 2.78% while inter-assay accuracy and precision was 97% and 4.11%.

**Comment #2**: It would be helpful to the reader to make sure to explain what abbreviation means the first time they use it (eg. IQR, CARDIA and so on).

**Response**: We have now clarified abbreviations of IQR on page 8 (line 180) and CARDIA on page 14 (line 267) are spelled out as it first appears in the paper.

**Comment #3**: I would suggest that it is important to provide all data or refer to the data availability.

**Response**: All relevant data are within the manuscript and a limited data set is privately available on Fig share and a doi has been issued (10.6084/m9.figshare.9913667)

**Comment #4**: It would be helpful to improve the resolution of figures.

**Response:** The resolution of figures is now improved and revised figures are uploaded.

**Comment #5:** In Table 1, how does activity level estimate?

**Response**: The methods used to quantify Leisure-time physical activity are described on page 7 (line number 145) of the revision.

**Comment #6**: In Figure 2, the minimum would start 0.

**Response**: Since a value of zero is arbitrary relative to the mean values of fasting glucose and the variability of the measure, we have chosen to center the figure Y axis on the mean value observed in the total population (mean=85 mg/dL) and to set the range as twice the standard deviation of fasting glucose (14) in this sample. This gives the reader a better perspective of glucose variability across TMAO groups relative to the size of glucose variability overall. This is now clarified in the figure legend.

**Reviewer # 2 Comments**

**General comments:** The manuscript explores the possibility of using Trimethylamine-N-oxide (TMAO), as an early biomarker of longitudinal glucose increase or prevalent impaired glucose regulation. Overall, the manuscript is well-written, and the study has several interesting information. But few errors with lack of validation makes the study weak. These needs to be addressed sufficiently to be considered for publication in Plos Journal.

**Response**: We appreciate the reviewer’s comments and have addressed each of the comments below.

**Comments # 1:** Plasma TMAO in Diabetes, whether TMAO is a cause or consequence of Diabetes (Pre-diabetes) are highly debatable. As several studies using TMAO and cardiovascular diseases provide contradicting results. Though this showed modest increase in the prevalence of prediabetes in a nonlinear fashion. Several factors which interfere with the conditions/ Pre-diabetes were lest discussed and also, Study validation is an absolute necessity.

**Response:** We agree that results from previous studies have mixed results in the association of TMAO and cardiovascular disease. We were able to replicate the null findings that were observed between TMAO and cross-sectional insulin resistance in the CARDIA study by Meyer et al*, the latter being a generally healthy population similar to ORIGINS. While our data cannot prove causality, they help to characterize TMAO in relation to early cardiometabolic risk which will help to inform the design and premise of future studies that can address causal associations with greater validity.

*Meyer KA, Benton TZ, Bennett BJ, Jacobs DR, Jr., Lloyd-Jones DM, Gross MD, et al. Microbiota-Dependent Metabolite Trimethylamine N-Oxide and Coronary Artery Calcium in the Coronary Artery Risk Development in Young Adults Study (CARDIA). Journal of the American Heart Association. 2016;5(10).

**Comment # 2:** There are no line numbers in the manuscript which makes it hard to pinpoint the errors.

**Response:** Line numbers are now added to facilitate the review

**Comment # 3:** 20-55 years are definitely not young population, Correct the sentences wherever needed (Eg: Last two lines of introduction).

**Response:** Reference to 20-55 years as young population was removed from the following lines (line 92 on page 5, line 318 and line 321 on page 16).

**Comment #4:** TMAO levels were modestly associated with increased age. Discuss more on this point.

**Response:** Table 1 shows the mean age of those in Tertile 3 is 36.01±1.03 compared to Tertile 1 which was 32.28±0.92. The reasons for this relationship are not entirely clear but one possibility is that older participants have decreased renal function leading to increased TMAO. In response to the reviewer’s comments, we have added more discussion on this point to the manuscript on page 14 as follows: “Renal function might also provide some level of explanation for discordant findings in our current data as compared to other cohorts. The fact that higher renal function increases TMAO clearance raises the strong potential for confounding. Specifically, it is possible that reduced renal function causes both elevated TMAO levels and clinical CVD events. Our observation of increased TMAO among older participants provides indirect support for this notion. As such, our adjustment for age in multivariable models helps to mitigate confounding by renal function although future studies with assessment of renal function will be important.”
