# Supplementary material for: Plasma Trimethylamine-N-oxide and impaired glucose regulation: Results from The Oral Infections, Glucose Intolerance and Insulin Resistance Study (ORIGINS)
Source: PLoS One. 2020 Jan 15;15(1):e0227482. doi: 10.1371/journal.pone.0227482 (PMC6961885; doi:10.1371/journal.pone.0227482)
Supplement: Attachment — Submitted filename: Response to Reviewers_11_27_19.docx [file pone.0227482.s005.docx]

We wish to thank the reviewer for the helpful comments, which have led to further strengthening of the manuscript.

**Reviewer #1 Comments**
Reviewer #1: I would suggest that the manuscript needs to provide all abbreviations used.
Page 3 line 6 CRP -> C-reactive protein
Page 5 line 105 FPG -> fasting plasma glucose (FPG)
Page 5 line 106 HbA1c -> hemoglobin A1c (HbA1c)
Page 6 line 124 fasting plasma glucose (FPG) -> FPG
Page 8 line 169 (PR) -> Delete
Page 8 line 182 Trimethylamine-N-oxide (TMAO) -> TMAO
Page 8 line 183 The Oral Infections, Glucose intolerance and Insulin Resistance Study (ORIGNS) -> ORIGINS
Page 11 line 211 hemoglobin A1c -> HbA1c
Page 11 line 212 Trimethylamine-N-oxide (TMAO) -> TMAO
Page 11 line 213 The Oral Infections, Glucose intolerance and Insulin Resistance Study (ORIGNS) -> ORIGINS
Page 14 line 276 CVD -> cardiovascular disease
Page 15 line 284 CKD -> chronic kidney disease
Page 15 line 301 Alternative Healthy Eating Index (AHEI) -> AHEI

It would be helpful to the reader to provide the rationale in the figure 2 legend which the authors explained in the response of comment 6.

**Response:** We appreciate the reviewer’s comments and have provided the expansion/acronyms as appropriate in the corresponding pages as noted below:

Page 3 line 68 CRP now reads as C-reactive protein (CRP)
Page 5 line 105 FPG now reads as fasting plasma glucose (FPG)
Page 5 line 106 HbA1c now reads as hemoglobin A1c (HbA1c)
Page 6 line 124 fasting plasma glucose is deleted and now reads as FPG
Page 8 line 169 (PR) is deleted
Page 8 line 182 Trimethylamine-N-oxide (TMAO) now reads as TMAO
Page 8 line 183 The Oral Infections, Glucose intolerance and Insulin Resistance Study (ORIGNS) now reads as ORIGINS
Page 11 line 211 hemoglobin A1c now reads as HbA1c
Page 11 line 212 Trimethylamine-N-oxide (TMAO) now reads as TMAO
Page 11 line 213 The Oral Infections, Glucose intolerance and Insulin Resistance Study (ORIGNS) now reads as ORIGINS
Page 14 line 276 CVD now reads as cardiovascular disease
Page 15 line 284 CKD now reads as chronic kidney disease
Page 15 line 301 Alternative Healthy Eating Index (AHEI) now reads as AHEI

The rationale that was explained in the comment 6 has been added to the figure 2. legend in the revised manuscript text.
